# 1-Point RANSAC UKF with Inverse Covariance Intersection for Fault Tolerance

**DOI:** 10.3390/s20020353

**Published:** 2020-01-08

**Authors:** Sun Young Kim, Chang Ho Kang, Jin Woo Song

**Affiliations:** 1 School of Intelligent Mechatronics Engineering, Sejong University, Seoul 05006, Korea; sykim77@sejong.ac.kr; 2 Department of Mechanical System Engineering, Kumoh National Institute of Technology, Gyeongbuk 39177, Korea; kcguri@kumoh.ac.kr

**Keywords:** fault tolerance, inverse covariance intersection, 1-point RANSAC UKF, robust estimation filtering

## Abstract

The fault tolerance estimation method is proposed to maintain reliable correspondences between sensor data and estimation performance regardless of the number of valid measurements. The proposed method is based on the 1-point random sample consensus (RANSAC) unscented Kalman filter (UKF), and the inverse covariance intersection (ICI)-based data fusion method is added to the update process in the proposed algorithm. To verify the performance of the proposed algorithm, two analyses are performed with respect to the degree of measurement error reduction and accuracy of generated information. In addition, experiments are conducted using the dead reckoning (DR)/global positioning system (GPS) navigation system with a barometric altimeter to confirm the performance of fault tolerance in the altitude. It is confirmed that the proposed algorithm maintains estimation performance when there are not enough valid measurements.

## 1. Introduction

Identifying reliable measurement data sequentially is most important for estimation algorithms because it is associated with estimation performance. Identification methods have been studied recently in References [1,2,3], which operate by checking the consistency of the data against the global model assumed to be generating the measurement data, and discarding as spurious any that do not fit into it. Among the identificaiton methods, random sample consensus (RANSAC) has been widely used as an effective outlier detection technique for robust estimation. However, because of the large amount of computation required to build the model, much research has been done to implement efficient algorithms, one of which is the combination of RANSAC and the Kalman filter structure [4,5,6,7]. There are some related approaches involving the combination of these concepts, and the aim of these methods is that the fitting process in the RANSAC begins again with an initial value obtained from the Kalman filter prediction and searches for inliers in the confidence interval [4]. Among these combination schemes, the 1-point RANSAC extended Kalman filter (EKF) is a recent technique that can efficiently operate algorithms [8,9]. Using the 1-point RANSAC method, assuming that probabilistic a priori information is available due to the Kalman filter, the sample size can be reduced to one point, and the number of hypotheses could also be reduced [8].

Although the efficiency of the fitting process in the RANSAC is improved by the assumption using the Kalman filter, there is a tendency whereby the consistency of the 1-point RANSAC EKF is much worse in certain situations. Inconsistency of the 1-point RANSAC EKF may occur particularly in situations where the measurement update process of the Kalman filter does not work properly. If there is no valid measurement determined by the 1-point RANSAC, then the Kalman filter will use the predicted value obtained by the time-update process in the filter without using the compensated value of the current measurements obtained by the measurement-update process in the filter. If this phenomenon continues in a dynamic situation, the predicted result of the filter may differ from the actual measured value, and the error of the initial value used in the RANSAC may increase. As a result, a fitting model with a large error is inevitably generated, and this error is propagated throughout the whole process of the 1-point RANSAC EKF.

In this paper, the proposed algorithm is based on the 1-point RANSAC EKF and has a similar filter structure to that of References [8,9]. However, the unscented Kalman filter (UKF) [10,11,12] is used as the main filter instead of the EKF in order to increase estimation efficiency in the nonlinear system. The UKF is a representative nonlinear filtering method that employs selected sigma points which are propagated through a system and measurement model. In the UKF, various algorithms of the UKF related to the proper positioning of the sigma points have been researched for reducing the unscented transform approximation error in dynamic situations [13,14,15]. In terms of points replacement, there are several alternative methods based on the particle filter [16,17,18,19] that are more flexible, as well as general algorithms for dealing with the fault tolerance estimation. However, since these algorithms have large computations, it has been decided to use the UKF in this paper.

In addition, error-bound analysis of the proposed algorithm is performed to evaluate the performance of measurement outlier elimination. Although this filter can guarantee the reliability of the measurement data, it may not have enough measurements to update the filter because of the evaluation process of RANSAC.

In case there are no good measurements after passing through the filter, we use the inverse covariance intersection (ICI) method [20] for fusing the estimated measurements obtained in the filter prediction process and the selected hypothesis model of the RANSAC iteration process, respectively. One of the well-known conservative fusion methods is covariance intersection (CI), which was introduced in Reference [21] and has been used in many developments and applications [22,23,24]. However, CI often provides fusion results that are too conservative for typical estimation tasks and communication networks, in general, preventing extreme correlation terms from occurring [20]. An alternate method is ellipsoidal intersection (EI) [25], which employs a common error term to model unknown correlations and reports a far less conservative result compared to CI. Although the consistency of EI has not been verified yet, it had been demonstrated to be effective in numerous applications [20]. Thus, an extension of EI is proposed that guarantees consistent fusion results in the presence of unknown common information, which is called ICI [20].

For that reason, in this paper, ICI is also used to generate the reliable measurements when there are no measurements satisfying the condition of the 1-point hypotheses evaluation process in the proposed algorithm because of measurement errors. As mentioned earlier, ICI guarantees consistent fusion results in the presence of unknown common information. In addition, its fusion results prove to be more accurate than measurement prediction results provided by the UKF when there are few valid measurements.

An outline of the paper is as follows. In Section 2, the whole process of 1-point RANSAC UKF is explained and its stability is also analyzed. The proposed method with ICI is introduced in Section 3. Subsequently, the ICI and its role in the proposed algorithm to improve the performance of the 1-point RANSAC UKF are discussed. In Section 4, experiments are performed using the dead reckoning (DR)/global positioning system (GPS) navigation system with a barometric altimeter to confirm the actual filter performance compared with conventional methods. The conclusions are summarized in Section 5.

## 2. 1-Point RANSAC UKF

In this section, we present the 1-point RANSAC UKF, which is a combination of RANSAC and UKF. The 1-point RANSAC UKF uses the available prior probabilistic information from the nonlinear filter, UKF, in the RANSAC model hypothesis stage, which allows the minimal sample size to be reduced to one, resulting in large computational savings.

### 2.1. 1-Point RANSAC and Its Filter Structure

The use of prior information can reduce the size of the data set that instantiates the model to the minimum size of one point. To generate the RANSAC hypothesis efficiently, prior information is obtained by the UKF. The whole process of the proposed 1-point RANSAC UKF with ICI is written in Algorithm 1.
**Algorithm 1** Pseudocode of 1-point RANSAC UKF with ICI.INPUT: $\left[x_{k-1}^{+},P_{k-1}^{+}\right]$ (k−1 step estimates), $T_{thr}$ (threshold)     p (probability of spurious-free hypothesis)     $n_{in}$ (number of inliers required)OUTPUT: $\left[x_{k}^{+},P_{k}^{+}\right]$ (k step estimates)**UKF prediction**1$\left[x_{k}^{-},P_{k}^{-},z_{k}^{-},S_{k}^{-}\right]$ = UKF_prediction $\left(x_{k-1}^{+},P_{k-1}^{+}\right)$**1-point hypothesis generation and evaluation**2for i=0 to $n_{hyp}$
3   $z_{i}$ = select_random_match $\left(z_{k}\right)$ [8]4   ${\tilde{x}}_{i}^{+}$ = UKF_state_update $\left(x_{k}^{-},z_{i}^{}\right)$ using Equation (15)5   ${\hat{z}}_{i}^{+}=h\left({\tilde{x}}_{i}^{+}\right)$: predicted measurements6   $z_{i}^{th}$ = find_matches_below_a_threshold $\left({\hat{z}}_{i}^{+},z_{k}^{},T_{thr}\right)$ [8]7   if size $size\left(z_{i}^{th}\right)>size\left(z_{i}^{inlier}\right)$ then8      
$z_{i}^{inlier}=z_{i}^{th}$
9      $\varepsilon =1-\frac{size\left(z_{i}^{inlier}\right)}{size\left(z_{k}^{}\right)},\;n_{hyp}=\frac{\log\left(1-p)\right)}{\log\left(1-\left(1-\varepsilon \right)\right)}$ [8]10   end if 11end for**UKF update**12if size $size\left(z_{i}^{inlier}\right)>n_{in}$ then13   $\left[x_{k}^{+},P_{k}^{+}\right]$ = UKF_update $\left(x_{k}^{-},z_{i}^{inlier}\right)$14else15   $\left[{\hat{z}}_{k}^{},{\hat{R}}_{k}^{}\right]$ = RANSAC_hypothesis_model $\left(z_{i}^{inlier},k\right)$
   $\left[{\hat{z}}_{k}^{+},{\hat{R}}_{k}^{+}\right]$ = ICI_data_fusion $\left[z_{k}^{-},S_{k}^{-},{\hat{z}}_{k}^{},{\hat{R}}_{k}^{}\right]$16   $\left[x_{k}^{+},P_{k}^{+}\right]$ = UKF_update $\left(x_{k}^{-},{\hat{z}}_{k}^{+}\right)$17end if

The first step of the 1-point RANSAC UKF is the UKF prediction process. The UKF makes use of the unscented transform (UT), which gives the Gaussian approximation to the filtering solutions of nonlinear optimal filtering problems [10,11,12]. To derive the process of the UKF, the system model f( ) and measurement model h( ) are considered as:

(1) $$x_{k+1}=f\left(x_{k}\right)+w_{k},$$

(2) $$z_{k}=h\left(x_{k}\right)+v_{k},$$

where $x_{k}\in R^{n}$ is the state variables, $z_{k}\in R^{m}$ is the measurement, $w_{k}\approx N\left(0,Q_{k}\right)$ is the Gaussian noise, and $v_{k}\approx N\left(0,R_{k}\right)$ is the Gaussian measurement noise. N( ) refers to Gaussian distribution, and $Q_{k},R_{k}$ are covariance matrices. To estimate the desired moments of the distribution of filter states, a matrix $\chi_{k-1}^{+}$ of 2n+1 sigma vector ${\left\{\chi \right\}}_{j}$ is defined by UT and written as follows [10]:

(3) $${\left\{\chi_{k-1}^{+}\right\}}_{0}=x_{k-1}^{+},$$

(4) $${\left\{\chi_{k-1}^{+}\right\}}_{j}=x_{k-1}^{+}+{\left(\sqrt{\left(n+\kappa \right)P_{x}{}_{k-1}^{+}}\right)}_{j},\;j=1,2,\ldots ,n,$$

(5) $${\left\{\chi_{k-1}^{+}\right\}}_{j}=x_{k-1}^{+}-{\left(\sqrt{\left(n+\kappa \right)P_{x}{}_{k-1}^{+}}\right)}_{j-n},\;j=n+1,\ldots ,2n,$$

where $x_{k-1}^{+}$ is the posterior estimation of $x_{k}$ at k−1 step, $P_{k-1}^{+}$ is the covariance matrix of $x_{k-1}^{+}$, and n is the dimension of $x_{k}$. The corresponding weights are

(6) $$\omega_{0}=\frac{\kappa }{n+\kappa },\;\omega_{j}=\frac{\kappa }{2\left(n+\kappa \right)}$$

Using sigma points and corresponding weights, the prediction step of the UKF is performed as:

(7) $$\chi_{k}^{-}=f\left(\chi_{k-1}^{+}\right),$$

(8) $$x_{k}^{-}=\sum_{j=0}^{2n}\omega_{j}{\left\{\chi_{k}^{-}\right\}}_{j},$$

(9) $$P_{k}^{-}=\sum_{j=0}^{2n}\omega_{j}\left[{\left\{\chi_{k}^{-}\right\}}_{j}-x_{k}^{-}\right]{\left[{\left\{\chi_{k}^{-}\right\}}_{j}-x_{k}^{-}\right]}^{T}$$

Measurement prediction value and its covariance are also estimated as:

(10) $$\zeta_{k}^{-}=h\left(\chi_{k}^{-}\right),$$

(11) $$z_{k}^{-}=\sum_{j=0}^{2n}\omega_{j}{\left\{\zeta_{k}^{-}\right\}}_{j},$$

(12) $$S_{k}^{-}=\sum_{j=0}^{2n}\omega_{j}\left[{\left\{\zeta_{k}^{-}\right\}}_{j}-z_{k}^{-}\right]{\left[{\left\{\zeta_{k}^{-}\right\}}_{j}-z_{k}^{-}\right]}^{T}$$

The second step of the 1-point RANSAC UKF is 1-point hypothesis generation and evaluation process (lines 2 to 11 in Algorithm 1) [8]. According to the principles of RANSAC, random state hypotheses ${\tilde{x}}_{i}^{+}$ are generated with a single match $z_{i}$ (line 3 in Algorithm 1), and data support ($z_{i}^{th}$) is computed by counting measurements inside the predefined threshold (line 6 in Algorithm 1). As the key difference compared with standard RANSAC, random hypotheses will be generated not only based on the measurements, but also on the predicted state variables $x_{k}^{-}$. Exploiting this prior knowledge leads to a reduction in the sample size necessary to express the model parameters from the minimal size to define the degrees of freedom of the model to only one data point.

The last step of the 1-point RANSAC UKF is the measurement update step with inliers ($z_{i}^{inlier}$). They are assumed to be generated by the true model, as they are at a small distance from the most supported hypothesis. Inliers are used in the measurement update step of the 1-point RANSAC UKF as follows (line 13 in Algorithm 1):

(13) $$P_{x,y}{}_{k}^{}=\sum_{i=0}^{2n}\omega_{i}\left[{\left\{\chi_{k}^{-}\right\}}_{i}-x_{k}^{-}\right]{\left[{\left\{\zeta_{k}^{-}\right\}}_{i}-z_{k}^{-}\right]}^{T},$$

(14) $$K_{k}=P_{x,y}{}_{k}^{}{\left(S_{k}^{-}\right)}^{-1},$$

(15) $$x_{k}^{+}=x_{k}^{-}+K_{k}\left(z_{k}-z_{k}^{-}\right),$$

(16) $$P_{k}^{+}=P_{k}^{-}-K_{k}S_{k}^{-}K_{k}^{T}$$

### 2.2. The Effect of the 1-point RANSAC UKF on Filter Convergence

In this section, analysis on the effect of the 1-point RANSAC UKF when there is a measurement error is performed based on the results of Reference [26]. In the previous work, [26], the error-bound value of the UKF was analyzed using the instrumental diagonal matrix. The error of state variables and measurements are defined, respectively.

(17) $${\bar{x}}_{k}^{-}=F_{k}{\bar{x}}_{k-1}^{+}+w_{k},$$

(18) $${\bar{z}}_{k}^{-}=H_{k}{\bar{x}}_{k}^{-}+v_{k},$$

where the estimation error is defined as ${\bar{x}}_{k}^{+}=x_{k}-{\hat{x}}_{k}^{+}{}_{k}$ (after measurement update of state variable estimation), and the prediction error is written as ${\bar{x}}_{k}^{-}=x_{k}-{\hat{x}}_{k}^{-}{}_{k}$. $F_{k}={\frac{\partial f\left(x\right)}{\partial x}|}_{x={\hat{x}}_{k}^{-}},H_{k}={\frac{\partial h\left(x\right)}{\partial x}|}_{x={\hat{x}}_{k}^{-}}$ are Jacobian matrices. To deal with the errors in the measurements, an instrumental diagonal matrix $\beta_{k}=diag\left\{\beta_{1},\beta_{2},\cdots ,\beta_{m}\right\}$ for the additional error of measurements are added to Equation (18) as follows, which reflects the effect of the additional unknown random constant error on the measurements [26].

(19) $${\bar{z}}_{k}^{}=\beta_{k}H_{k}{\bar{x}}_{k}^{-}+v_{k}$$

If there is the random constant error $b_{k}$ in the actual measurement,

(20) $$z_{k}^{}=H_{k}x_{k}^{}+b_{k}+v_{k}$$

Thus, the measurement error ${\bar{z}}_{k}$ can be expressed as:

(21) $${\bar{z}}_{k}=z_{k}-{\hat{z}}_{k}^{-}=H_{k}x_{k}+b_{k}+v_{k}-H_{k}{\hat{x}}_{k}^{-}=H_{k}{\bar{x}}_{k}^{-}+b_{k}+v_{k}$$

By using the equality of Equations (19) and (21), $\beta_{k}$ can be expressed as:

(22) $$\beta_{k}=I+b_{k}{\left(H_{k}{\bar{x}}_{k}^{-}\right)}^{T}{\left(H_{k}{\bar{x}}_{k}^{-}{\bar{x}}_{k}^{-}{}^{T}H_{k}^{T}\right)}^{-1},$$

where I is the identity matrix. According to Equation (22), the relationship between $\beta_{k}$ and $b_{k}$ is expressed with the prediction error. For convenient analysis of the stability of the 1-point RANSAC UKF, approaches used in the previous work [26] are employed in order to simplify the error expression. In addition, some assumptions are held for verifying the boundedness of the estimation errors in the 1-point RANSAC UKF:

There exist real constants which are related to the system model and measurement model written in Equations (1) and (2):

(23) $$\begin{array}{l} system\;matrix:f_{\min,\;}f_{\max\;}>0\\ measurement\;matrix:h_{\min,\;}h_{\max}>0\\ covariance\;matrix:q_{\min,\;}q_{\max,\;}p_{\min,\;}p_{\max,\;}R_{\max}>0\\ error\;vector:b_{\max}>0 \end{array}$$

such that the following bounds on matrices of filter models are satisfied for every time index k as follows:

(24) $$f_{\min}^{2}I\leq F_{k}F_{k}^{T},\;h_{\min}^{2}I\leq H_{k}H_{k}^{T},\;\left\|F_{k}\right\|\leq f_{\max},$$

(25) $$\left\|H_{k}\right\|\leq h_{\max},\;q_{\min}I\leq Q_{k}^{*},\;R_{k}\leq R_{\max}I$$

The boundary of $\beta_{k}$ is

(26) $$\left\|\beta_{k}\right\|\leq \sqrt{m}\left|1+\frac{b_{\max}\sqrt{p_{\max}}}{h_{\max}p_{\max}}\right|=\beta_{\max},$$

where ‖ ‖ is the matrix norm. The boundary of $\beta_{k}$ is based on Equation (22). The predicted error covariance matrix of state variables $P_{k}^{-}$ is defined as follows:

(27) $${\hat{P}}_{k}^{-}=\left[F_{k}\left(I-K_{k}\beta_{k}H_{k}\right)\right]{\hat{P}}_{k-1}^{-}{\left[F_{k}\left(I-K_{k}\beta_{k}H_{k}\right)\right]}^{T}+Q_{k}^{*},$$

where $Q_{k}^{*}=Q_{k}^{}+F_{k}K_{k}R_{k}{\left(F_{k}K_{k}\right)}^{T}+\delta P_{k}$, and $\delta P_{k}$ refers to the error between ideal error covariance matrix and unscented transformed error covariance matrix.

The weighted error square of state variables is defined as:

(28) $$e_{k}\left({\bar{x}}_{k}^{-}\right)={\left({\bar{x}}_{k}^{-}\right)}^{T}{\left({\hat{P}}_{k}^{-}\right)}^{-1}{\bar{x}}_{k}^{-}$$

According to the results of Reference [26], the expectation of the weighted error square is bounded as:

(29) $$E\left\{e_{k}\left({\bar{x}}_{k}^{-}\right)|{\bar{x}}_{k}^{-}\right\}\leq \frac{K_{\max}^{2}f_{\max}^{2}q_{\max}^{}n}{p_{\min}}+\frac{R_{\max}^{}m}{p_{\min}}+{\left[1+\frac{q_{\min}^{}I}{{\left(f_{\max}^{}+f_{\max}^{}K_{\max}^{}\beta_{\max}^{}h_{\max}\right)}^{2}-P_{\max}^{}}\right]}^{-1}e_{k}\left({\bar{x}}_{k}^{-}\right)$$

If the coefficient of $e_{k}\left({\bar{x}}_{k}^{-}\right)$ is replaced with 1−λ and the last term is substituted with an arbitrary positive value μ, Equation (28) can be rewritten as:

(30) $$\begin{array}{l} E\left\{e_{k}\left({\bar{x}}_{k}^{-}\right)|{\bar{x}}_{k-1}^{-}\right\}\leq \left(1-\lambda \right)e_{k}\left({\bar{x}}_{k}^{-}\right)+\mu \\ E\left\{e_{k}\left({\bar{x}}_{k}^{-}\right)|{\bar{x}}_{k-1}^{-}\right\}-e_{k}\left({\bar{x}}_{k}^{-}\right)\leq -\lambda e_{k}\left({\bar{x}}_{k}^{-}\right)+\mu \end{array}$$

Finally, applying the Lemma of Reference [27] to Equation (30), the stochastic process of ${\bar{x}}_{k}^{-}$ is bounded. In Reference [27], an analysis of the error-bound value of the EKF when given the stochastic system was performed. The inequality formula in a form such as Equation (30) was used to determine the filter convergence of the estimation error, and the detailed verification process was written. Therefore, it is shown that the estimation error of the proposed algorithm remains bounded if the filter system satisfies some assumptions listed in Equations (23)–(26). If there is an error in the measurement, the 1-point RANSAC UKF has an inlier detection process (1-point hypotheses evaluation) that confirms the quality of the measurement, which can cause the $\beta_{k}$ value to have less impact on the estimation performance than the conventional UKF.

Thus, as shown in Equation (29), the 1-point RANSAC UKF has a smaller estimation error range of the state variable than the general UKF ($\beta_{\max}$ can be reduced to $\sqrt{R_{\max}}$ in the case of the 1-point RANSAC UKF).

However, the proposed filter cannot improve the estimation performance if there is an insufficient number of measurements or there is no measurable value after passing the 1-point hypotheses evaluation process. In this paper, Kullback-Leibler divergence (KLD) is used to set the reference value of the valid measurement number written in line 12 of Algorithm 1.

The required number $n_{in}$ of samples is determined with probability 1−δ and KLD between sample-based maximum likelihood estimate and the true distribution (desired distribution), which is less than a threshold ε as follows [28,29,30]:

(31) $$n_{in}=\frac{n_{p}-1}{2\varepsilon }{\left(1-\frac{2}{9\left(n_{p}-1\right)}+\sqrt{\frac{2}{9\left(n_{p}-1\right)}}z_{1-\delta }\right)}^{3},$$

where $z_{1-\delta }$ is the upper quantile of the standard normal distribution. The optimal value of $z_{1-\delta }$ can be founded in standard statistical tables for the typical value of δ, and $n_{p}$ is the number of bins with support [28,30]. The result of Equation (31) gives the effective number of samples needed to approximate a discrete distribution with an upper bound *ε* on the KLD.

To get enough numbers of measurements, the previous work [8] increased the threshold value of the discrimination process and performed the measurement update of the filter with the measurements containing a relatively small error. However, this approach may cause a larger estimation error depending on the measurement error magnitude compared to only using the prediction process of the filter.

Thus, when the number of valid measurements is insufficient, a method of generating artificial measurements using past collected measurements and measurement models was studied. In this paper, the ICI method is applied to the proposed algorithm to get valid measurements without increasing the estimation error.

## 3. Inverse Covariance Intersection

In this section, we prove that the estimation performance using the fusion method based on ICI is better than the case where only the filter prediction process is performed when there are no good measurements.

### 3.1. ICI-Based Data Fusion Method in the Measurement Update Process

A consistent combination of the estimates $\left(z_{k}^{-},\;S_{k}^{-}\right)$ obtained in the filter prediction process and $\left({\hat{z}}_{k},\;{\hat{R}}_{k}\right)$ obtained in the selected hypothesis model of the RANSAC iteration process is provided by $\left({\hat{z}}_{k}^{+},\;{\hat{R}}_{k}^{+}\right)$ with ICI as follows [20]:

(32) $${\hat{z}}_{k}^{+}=C_{R}{\hat{z}}_{k}^{}+C_{P}{\hat{z}}_{k}^{-},$$

(33) $${\left({\hat{R}}_{k}^{+}\right)}^{-1}={\left({\hat{R}}_{k}\right)}^{-1}+{\left(S_{k}^{-}\right)}^{-1}-{\left(\gamma {\hat{R}}_{k}+\left(1-\gamma \right)S_{k}^{-}\right)}^{-1},$$

for any γ∈[0,1]. The gains in Equation (32) are set as:

(34) $$C_{R}={\hat{R}}_{k}^{+}\times \left({\left({\hat{R}}_{k}\right)}^{-1}-\gamma {\left(\gamma {\hat{R}}_{k}+\left(1-\gamma \right)S_{k}^{-}\right)}^{-1}\right),$$

(35) $$C_{P}={\hat{R}}_{k}^{+}\times \left({\left(S_{k}^{-}\right)}^{-1}-\left(1-\gamma \right){\left(\gamma {\hat{R}}_{k}+\left(1-\gamma \right)S_{k}^{-}\right)}^{-1}\right),$$

where ${\hat{R}}_{k}$ refers to the fitting quality of the selected hypothesis model. In general, it is calculated as the mean square error between model values and measurements. Finally, by using estimate measurement (${\hat{z}}_{k}^{+}$), the process of the UKF measurement update is performed when there is not enough measurement compared with $n_{in}$ as shown in Algorithm 1 (lines 14 to 17).

### 3.2. Performance Analysis of ICI Compared with Filter Predicted Value

To verify that using ICI-based fusion method is better than using only the filter prediction process in the case that there are no good measurements after passing through the filter, a simple analysis is performed.

Comparison of the two covariances is explained as:

(36) $${\left({\hat{R}}_{k}^{+}\right)}^{-1}-{\left(S_{k}^{-}\right)}^{-1}={\left({\hat{R}}_{k}\right)}^{-1}-{\left(\gamma {\hat{R}}_{k}+\left(1-\gamma \right)S_{k}^{-}\right)}^{-1}$$

Equation (36) is converted into a diagonal matrix form for ease of analysis. Multiply the transformation matrix $T_{k}^{}$ on both sides of components in Equation (36) to convert into a diagonal matrix as follows:

(37) $${\bar{D}}_{k}=T_{k}^{}\left({\left({\hat{R}}_{k}^{+}\right)}^{-1}-{\left(S_{k}^{-}\right)}^{-1}\right)T_{k}^{T}={\left({\hat{D}}_{k}^{}\right)}^{-1}-{\left(\gamma {\hat{D}}_{k}^{}+\left(1-\gamma \right)D_{k}^{-}\right)}^{-1},$$

where $D_{k}=T_{k}P_{k}T_{k}^{T}$ is the diagonal matrix, and $T_{k}^{}$ is a transformation matrix which can be computed with the aid of an eigenvalue decomposition as in Reference [26]. If an i-th diagonal component of $D_{k}$ is expressed as ${\left(d_{k}\right)}_{i}={\left(D_{k}\right)}_{ii}$, the diagonal entries are

(38) $${\left({\bar{D}}_{k}\right)}_{jj}=\frac{1}{{\left({\hat{d}}_{k}^{}\right)}_{j}}-\frac{1}{\gamma {\left({\hat{d}}_{k}^{}\right)}_{j}+\left(1-\gamma \right){\left(d_{k}^{-}\right)}_{j}}=\left(\frac{1-\gamma }{\gamma {\left({\hat{d}}_{k}^{}\right)}_{j}+\left(1-\gamma \right){\left(d_{k}^{-}\right)}_{j}}\right)\times \left(\frac{{\left(d_{k}^{-}\right)}_{j}}{{\left({\hat{d}}_{k}^{}\right)}_{j}}-1\right)$$

The first part of Equation (38), $\frac{1-\gamma }{\gamma {\left({\hat{d}}_{k}^{}\right)}_{j}+\left(1-\gamma \right){\left(d_{k}^{-}\right)}_{j}}$ is always positive under the given conditions: $\gamma >0,{\left({\hat{d}}_{k}^{}\right)}_{j}>0,{\left(d_{k}^{-}\right)}_{j}>0$. According to Equation (12), $S_{k}^{-}$ includes prediction error of state variables and measurement noise covariance. Thus, ${\left(d_{k}^{-}\right)}_{j}$ always satisfies ${\left(d_{k}^{-}\right)}_{j}\geq {\left(T_{k}R_{k}T_{k}^{T}\right)}_{j}$. In the case of ${\hat{R}}_{k}$, it is based on the goodness of fit and the error of the best hypothesis model is always under the measurement error. Thus, ${\left({\hat{d}}_{k}^{}\right)}_{j}\leq {\left(T_{k}R_{k}T_{k}^{T}\right)}_{j}$. Finally, by using two properties, ${\left({\bar{D}}_{k}\right)}_{jj}\geq 0$. This means that ${\hat{R}}_{k}^{+}$ is always smaller than $S_{k}^{-}$. Therefore, the ICI-based fusion method can generate measurements which have a smaller error than the predicted measurements obtained based on the prediction of the filter.

### 3.3. Simulations to Verify the Performance of ICI

In this section, the principle of the proposed algorithm is explained through a one-dimensional example, and the ICI performance is also verified. To explain the advantage of the proposed algorithm, the univariate nonstationary growth model (UNGM) is used in this paper. The UNGM has been used as a benchmark model in several previous works [31,32] due to its highly nonlinear and bimodal properties.

The dynamic state space and the measurement model of UNGM can be written as:

(39) $$x_{k}=\alpha x_{k-1}+\beta \frac{x_{k-1}}{1+x_{k-1}^{2}}+\gamma \cos\left(1.2\left(k-1\right)\right)+w_{n},$$

(40) $$z_{k}=\frac{x_{k}^{2}}{20}+v_{n},\;n=1,2,\cdots N_{ex},$$

where $w_{n}\approx N\left(0,1\right)$, $v_{n}\approx N\left(0,1\right)$ (Gaussian distributions). In this example, α=1, β=15, γ=0.1, and $x_{0}=10,$ $P_{0}=1,$ $N_{ex}=200$.

**Figure 1:**
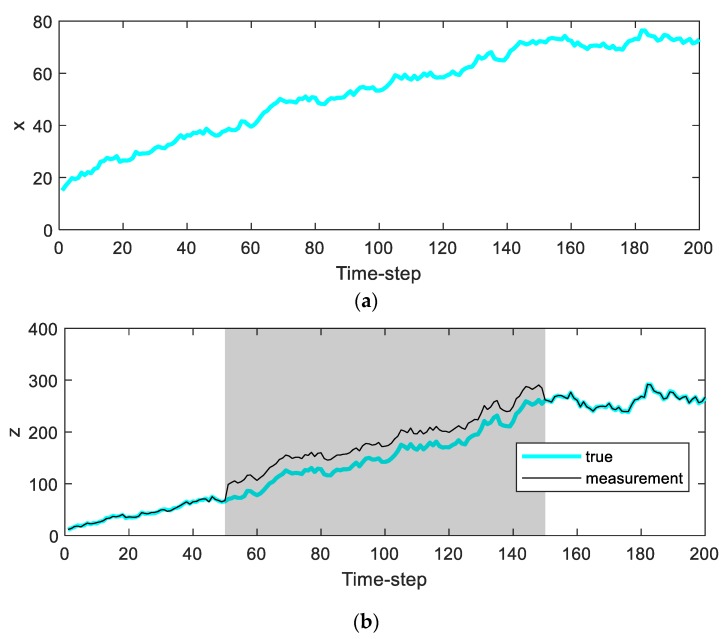
True trajectories of the simulation: (**a**) the true value of $x_{n}$ in the simulation; (**b**) the true value of $z_{n}$ and $z_{n}$ with bias error in the gray-colored period.

In this example, a bias type error is inserted in a specific interval (50 to 150 time-step of simulations) of the measurement values to show the difference in performance between the proposed algorithm based on ICI and the conventional techniques (UKF, 1-point RANSAC UKF), which are described in Figure 1. The 1-point RANSAC UKF has the same filter structure as the 1-point RANSAC EKF, except that the filter update process in the 1-point RANSAC UKF uses unscented transform. The purpose of this simulation is not to analyze state variable estimates due to nonlinearity, but to compare performance to reduce the effects of measurement error. Thus, the comparison result of 1-point RANSAC EKF is not included.

The insertion of a bias type error is intended to assume that there is no valid measurement through the RANSAC process during that interval. The magnitude of the bias error is larger than the threshold for distinguishing the outliers in the RANSAC process ($T_{thr}$) [8] and is set to 30 in this example.

**Figure 2:**
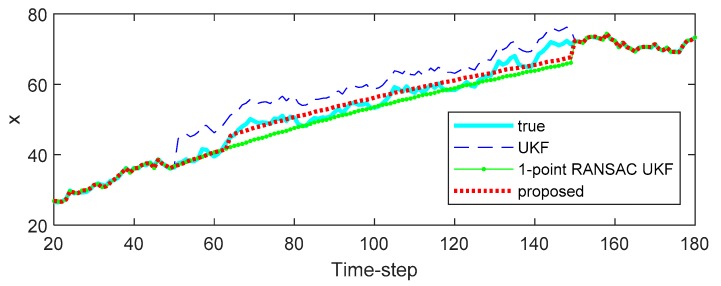
The estimation results of the simulation.

**Figure 3:**
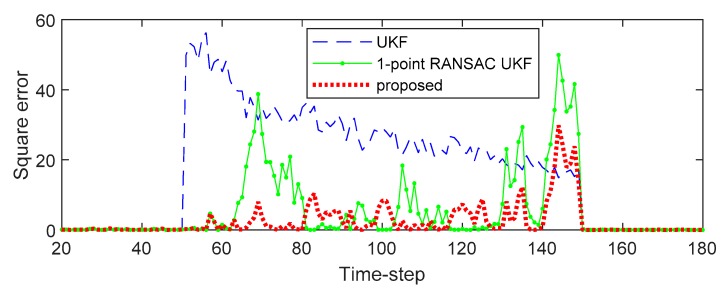
The square error of the simulation.

Figure 2 and Figure 3 show the effect of bias error on the estimated state variables of each filter. Figure 2 shows the estimated state variables, and Figure 3 shows the square error of the state variable. As shown in the results, the UKF propagates the measurement error directly to the state variables. In the case of the 1-point RANSAC UKF, the bias error is completely eliminated by the RANSAC process, but there is no effective measurement value, so the estimation error can occur because the measurement update process of the filter cannot be performed and only the prediction process by the system model is performed (the uncertainty of the estimated result has increased). On the other hand, the proposed algorithm integrates the estimated measurement value obtained from the measurement model (${\hat{z}}_{k}^{}$) with predicted values obtained from the time update process of the filter ($z_{k}^{-}$) using ICI. Since the combined value (${\hat{z}}_{k}^{+}$) is ultimately used as the measurement value, the proposed technique can have a relatively small state estimation error even when there is no valid measurement value (in the presence of bias error). Finally, Table 1 shows the root mean square error (RMSE) of the 500 Monte-Carlo simulation results including bias error in the measurement.

However, since the performance of the proposed algorithm is affected by the quality and number of the measurements constituting the measurement model, it is important to acquire the effective measurements at an initial period. The proper number of measurements should be selected to construct an accurate measurement model, and in this simulation, we design the initial measurement model with the initial 50 measurements.

## 4. Experiments

The experiments are conducted using a barometric altimeter to confirm the actual filter performance. The system model is set the same as the model of the previous paper [33]. The system is called the 10th order DR/GPS navigation system, and the state variables of the filter are position error ($\delta P_{N},\;\delta P_{E},\;\delta P_{D}$) in the NED frame, odometer scale factor error (δs), attitude error ($\varphi_{N},\;\varphi_{E},\;\varphi_{D}$) in the NED frame, and gyro bias ($\varepsilon_{x},\;\varepsilon_{y},\;\varepsilon_{z}$) [33]. The MEMS IMU (SMI 130, Bosch, Gerlingen, Germany)-based DR/GPS system was proposed, and the DR/GPS system is expanded from 2D to 3D positioning of the vehicle. Thus, altitude is additionally estimated with GPS and the barometric altimeter (BMP 280, Bosch). In this system, the inertial sensor SMI 130 (Bosch) is installed in the interior of the vehicle. Specifications of the sensors are summarized in Table 2. For the attitude and gyro calibration, the vehicle remained stationary for the first 10 minutes, then drove the oval-shaped park four times for the initial calibration and alignment process of the IMU [33].

The system model and the system matrix $F_{k}$ of the DR/GPS navigation system are expressed by

(41) $$x_{k+1}=F_{k}x_{k}+w_{k},$$

(42) $$F_{k}=\left[\begin{array}{cccc} O_{3\times 3} & C_{b}^{n}\left[\begin{array}{c} u_{odo}\\ 0\\ 0 \end{array}\right] & \left(C_{b}^{n}\left[\begin{array}{c} su_{odo}\\ 0\\ 0 \end{array}\right]\times \right) & O_{3\times 3}\\ O_{1\times 3} & -\frac{1}{\tau } & O_{1\times 3} & O_{1\times 3}\\ F_{31} & O_{3\times 1} & F_{33} & -C_{b}^{n}\\ O_{3\times 3} & O_{3\times 1} & O_{3\times 3} & O_{3\times 3} \end{array}\right],$$

where $O_{m\times n}$ refers to the m×n zero matrix. $u_{odo}$ and s are odometer output and odometer scale factor, respectively. $C_{b}^{n}$ is a direction cosine matrix from the body frame to the navigation frame, and τ is the time constant. [•]× is a skew-symmetric matrix representing the vector cross product by the vector [•]. In addition, its submatrices ($F_{31},\;F_{33}$) are expressed by [33,34]

(43) $$\begin{array}{l} F_{31}=\left[\begin{array}{ccc} \frac{1}{R_{m}+h}(\Omega_{D}-\frac{\rho_{N}R_{tt}}{R_{t}+h}) & 0 & -\frac{\rho_{N}}{R_{t}+h}\\ -\frac{\rho_{E}R_{mm}}{{\left(R_{m}+h\right)}^{2}} & 0 & -\frac{\rho_{E}}{R_{m}+h}\\ \frac{1}{R_{m}+h}(-\Omega_{N}-\rho_{N}\sec^{2}L-\frac{\rho_{D}R_{tt}}{R_{t}+h}) & 0 & -\frac{\rho_{D}}{R_{t}+h} \end{array}\right]\;\\ F_{33}=\left[\begin{array}{ccc} 0 & \Omega_{D}+\rho_{D} & -\rho_{E}\\ -\Omega_{D}-\rho_{D} & 0 & \Omega_{N}+\rho_{N}\\ \rho_{E} & -\Omega_{N}-\rho_{N} & 0 \end{array}\right] \end{array}$$

where L and h are the position in latitude and height, and subscript N, E, D represent the north, east, and vertical down direction in the navigation frame, respectively. The additional parameters written in Equation (43) can be summarized as follows [34]:

(44) $${\left[\begin{array}{ccc} \rho_{N} & \rho_{E} & \rho_{D} \end{array}\right]}^{T}={\left[\begin{array}{ccc} \frac{V_{E}}{R_{t}+h} & -\frac{V_{N}}{R_{m}+h} & -\frac{V_{E}\tan L}{R_{t}+h} \end{array}\right]}^{T},$$

(45) $${\left[\begin{array}{ccc} \Omega_{N} & 0 & \Omega_{D} \end{array}\right]}^{T}={\left[\begin{array}{ccc} \Omega \cos L & 0 & -\Omega \sin L \end{array}\right]}^{T},$$

(46) $$\begin{array}{l} R_{m}=\frac{R_{0}(1-e^{2})}{{\left(1-e^{2}\sin^{2}L\right)}^{3/2}}\;\\ R_{mm}=\frac{\partial R_{m}}{\partial L}=\frac{3R_{0}(1-e^{2})e^{2}\sin L\cos L}{{\left(1-e^{2}\sin^{2}L\right)}^{5/2}} \end{array}$$

(47) $$\begin{array}{l} R_{t}=\frac{R_{0}}{{\left(1-e^{2}\sin^{2}L\right)}^{1/2}}\;\\ R_{tt}=\frac{\partial R_{t}}{\partial L}=\frac{R_{0}e^{2}\sin L\cos L}{{\left(1-e^{2}\sin^{2}L\right)}^{3/2}} \end{array}$$

where V is the velocity of the vehicle. $R_{0}$ is the radius of the Earth, and e is the major eccentricity of the Earth. Ω is the Earth’s rate with respect to the inertial frame.

The measurement update for attitude obtained from the accelerometer ($\phi_{acc},\;\theta_{acc}$) and barometric altitude ($h_{baro}$) is performed in order to compensate for navigation errors. Measurements and the matrix of measurement update model are defined as:

(48) $$z_{k}=\;{\left[\begin{array}{ccc} \phi_{DR} & \theta_{DR} & P_{D,}{}_{DR} \end{array}\right]}^{T}-{\left[\begin{array}{ccc} \phi_{acc} & \theta_{acc} & h_{baro} \end{array}\right]}^{T}=H_{k}x_{k}+v_{k},$$

(49) $$H_{k}=\left[\begin{array}{ccc} O_{1\times 4} & \left[\begin{array}{ccc} -\frac{\cos\psi }{\cos\theta } & -\frac{\sin\psi }{\cos\theta } & 0 \end{array}\right] & O_{1\times 3}\\ O_{1\times 4} & \left[\begin{array}{ccc} \sin\psi & -\cos\psi & 0 \end{array}\right] & O_{1\times 3}\\ \left[\begin{array}{cccc} 0 & 0 & 1 & 0 \end{array}\right] & O_{1\times 3} & O_{1\times 3} \end{array}\right],$$

where $\phi_{DR}$ and $\theta_{DR}$ are the roll and pitch angle obtained from the pure navigation output, respectively. $P_{D,}{}_{DR}$ is the height obtained from the pure navigation output. $\phi_{acc}$ and $\theta_{acc}$ are also the roll and pitch angle calculated by the accelerometer [35,36]. ψ is the yaw angle, and θ is the pitch angle of the target system [33]. $O_{i\times j}$ represents the i×j zero matrix.

Barometric altimeters sense altitude by measuring the change in aerostatic pressure accompanying a change in altitude [37,38]. The conversion of measured air pressure to altitude is based on a theoretical standard atmosphere and the assumption that air is an ideal gas. The altitude equation is written as:

(50) $$h_{baro}=\frac{T_{0}}{\lambda_{p}}\left[1-{\left(\frac{P_{m}}{P_{0}}\right)}^{\lambda R_{g}/g}\right],$$

where $T_{0}$ is the temperature at sea level, and $P_{0}$ refers to pressure at sea level. In addition, $P_{m}$ is the pressure measurement at the altitude, $R_{g}$ is the universal gas constant, g is local gravity, and $\lambda_{p}$ is the lapse rate [37,38]. If GPS signals are not received during the vehicle test, the altitude measurement will only depend on the barometric altimeter and it will be vulnerable to changes in atmospheric pressure ($P_{m}$) as shown in Equation (50). This situation occurs frequently when the vehicle goes down or when climbing in the underground parking lot, regardless of the speed of the vehicle.

**Figure 4:**
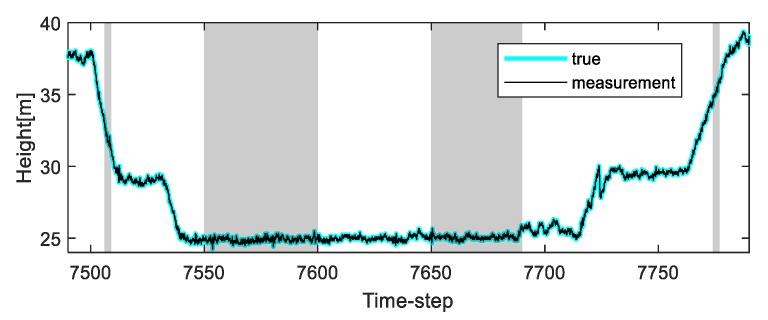
The true altitude trajectory and measurements of the underground parking lot under normal conditions (four shaded sections: generating measurement error).

To solve this problem, we apply the proposed algorithm to this system. Vehicle tests were carried out in the underground parking lot in the GPS outage area, and the altitude trajectory is shown in Figure 4. The average speed of the vehicle was maintained at 30 km/h. Experimental analysis was carried out only on the altitude value to which the proposed algorithm was applied. In the one case of several experiments, the altitude change was measured when the car came down the ramp, and the measurement error occurred during the operation due to the atmospheric pressure change (7507 to 7508 time-step, 7550 to 7600 time-step, 7650 to 7690 time-step, and 7775 to 7776 time-step, as shown in Figure 4). In addition, the true value of the altitude was determined by the values obtained from the terrain information database and the integrated navigation system (Trimble DR + GPS, performance characteristic of the system: less than 3 m altitude error).

**Figure 5:**
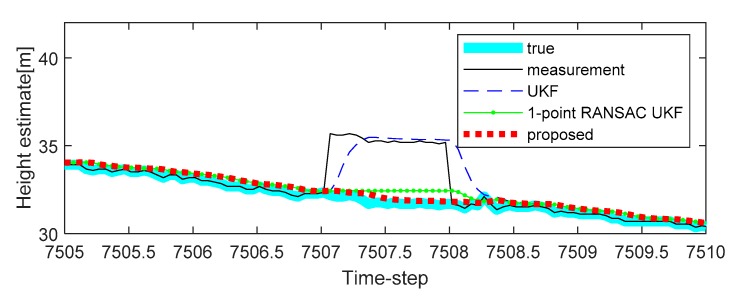
The first experimental results of height estimation with bias type measurement error in the first period (7507 to 7508 time-step): case 1.

In Figure 5, the estimated height of the proposed algorithm is shown, and the estimated heights of the target system obtained by conventional algorithms are also shown for comparison. It is confirmed that the proposed algorithm has better estimate performance compared with conventional algorithms because of 1-point RANSAC and ICI-based data fusion when there is an error in the measurement. In the case of the UKF, this filter does not isolate the measurement disturbance, so the effect of measurement disturbance is reflected in the height estimation result. Whereas, applying the RANSAC algorithm to the UKF can alleviate errors due to measurement disturbances. Finally, to make the estimation performance better (a reflection of the tendency to altitude change with time), measurements should be generated using ICI when there is no valid measurement (7507 to 7508 time-step as shown in Figure 5).

**Figure 6:**
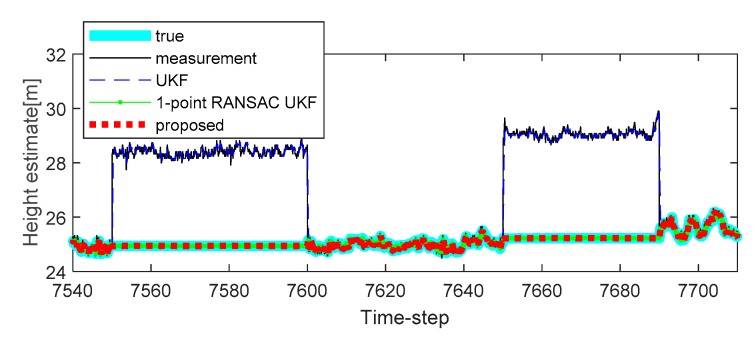
The second experimental results of height estimation with random bias type measurement errors in the second (7550 to 7600 time-step) and third period (7650 to 7690 time-step): case 2.

**Figure 7:**
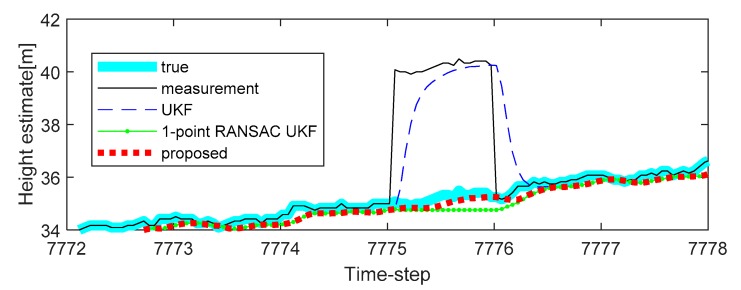
The last experimental results of height estimation with bias type measurement error in the last period (7775 to 7776 time-step): case 3.

Additional experiments were conducted to verify the performance of the proposed method on different trajectories. The second experiment reflected a scenario in which a sudden change in the atmospheric pressure occurs continuously in the barometric altimeter. The experimental results are shown in Figure 6, and it is confirmed that the compensation performance of the proposed method is less improved when compared with the 1-point RANSAC UKF in cases where there is no altitude change through Figure 6 (the sudden changes of the measurement caused by the atmospheric pressure are 3.5 m and 4 m). In this case, the performance of altitude error compensation is sufficient even when the 1-point RANSAC UKF is used. However, when the atmospheric pressure changes with altitude change, as shown in Figure 7 (also shown in Figure 5), we can confirm that the proposed algorithm has the best altitude error compensation. In this experiment, the altitude error caused by a sudden change in the atmospheric pressure is 5 m, which is larger than the discrimination error of the building floor (3.5 m). Lastly, Table 3 shows the RMSE of the 10 experiments results, including the measurement error of each case. It is confirmed that the proposed algorithm has the smallest error when compared with existing methods. For the same reason as in the previous simulations, the experiments do not include the performance comparison results of the 1-point RANSAC EKF.

## 5. Conclusions

In this paper, a 1-point RANSAC UKF with ICI-based data fusion method was proposed and analyzed from two perspectives: reduction of measurement error affecting estimated accuracy and accuracy of generated information when there were no valid measurements. According to these analyses, it was confirmed that the proposed method could maintain estimated performance regardless of the number of valid measurements. In addition, experiments were conducted using the DR/GPS system with the barometric altimeter to confirm the compensation performance of the proposed algorithm. The experimental results showed that the altitude error caused by sudden pressure changes was compensated well compared with the conventional algorithms when there was a height change of the vehicle.

## Figures and Tables

**Table 1:** RMSE of simulations.

UKF	1-Point RANSAC UKF	1-Point RANSAC UKF with ICI (Proposed)
3.7655	2.1152	1.4265

**Table 2:** Specification of the sensors.

	SMI 130 Gyro	SMI 130 Accelerometer	BMP 280 Barometer
Zero-point offset	±1 deg/s	±70 mg	-
Offset variation	±1 deg/s	±65 mg	-
Pressure range	-	-	300~1100 hPa
RMS noise	$0.02\;\deg/s/\sqrt{Hz}$	0.19 $mg/\sqrt{Hz}$	1.3 Pa
Sampling rate	20 Hz	20 Hz	20 Hz

**Table 3:** RMSE of experiments.

Case	UKF	1-Point RANSAC UKF	1-Point RANSAC UKF with ICI (Proposed)
1	0.9212	0.3509	0.2625
2	2.1919	0.3105	0.3071
3	1.0004	0.4468	0.3281
